# On a class of bimodal oscillations powered by a steady, zero-frequency force—Implications to energy conversion and structural stability

**DOI:** 10.1073/pnas.2311412120

**Published:** 2023-09-11

**Authors:** Amnon Zalman Yariv

**Affiliations:** ^a^Department of Applied Physics, California Institute of Technology, Pasadena, CA 91125; ^b^Department of Electrical Engineering, California Institute of Technology, Pasadena, CA 91125

**Keywords:** oscillation, parametric, bimodal, energy conversion, structural stability

## Abstract

I propose that there exists in natural and artificial environments a class of resonant oscillations that can be excited directly by a steady, zero-frequency force such as that of wind, water, electric field. A member of this class comprises two normally independent oscillating modes of a system, for example, a building or bridge, which, separately, cannot be driven by a zero-frequency force. Agreeing on terms of collaboration, the two modes engage in a joint oscillation powered by the steady zero-frequency force in which they drive each other, one directly and the other parametrically. I observed a bimodal vibration belonging to this class in a home shower where the two modes are the pendulum excursion and the torsional twisting of a freely suspended showerhead which break into a joint oscillation above a threshold value of the water flow rate. I advance a theoretical model which predicts and explains the main features of the observations. The model constitutes an extension to two modes of a proposal and demonstration in 1883 by Lord Rayleigh and Michael Faraday for the excitation of a single resonant mode by modulating a system parameter at twice the resonance frequency. The proposal is credited with the launching of parametric physics. The Experiments section of this report consists of three linked video clips photographed in the home shower which support the basic theoretical assumptions. The ubiquity of zero-frequency forces, such as that of wind, and their direct conversion to alternating on-resonance system vibrations endows the class with an amplified destructive potential with implications for structural stability.

## Significance

I view the main importance of my work in its impact on the following three areas: a category of modes in man-made and in natural environments, implications to energy harvesting, for example—direct DC to AC conversion in hydromechanical systems, implications to structural stability (bridge and building collapse). The abstract and introduction to the article provide a more detailed description of the impact and implications of the proposed mode of oscillation.

Oscillations are inextricably woven into the fabric of physics as well as into our daily experienc. Most involve a “system” which is driven by a force oscillating at a frequency near or at that of a resonance of the system. Typical examples are a child pushed by a parent on a swing, the quantum mechanical wave functions of electrons in atoms, and optical resonator modes. A second excitation method, proposed by Lord Rayleigh and demonstrated by Michael Faraday (1), is to drive an oscillation by modulating a parameter of the system (i.e., parametrically) at a frequency which is double that of the excited resonance. An example would be that of the older child on the swing, this time without a parent, powering its own swinging by pulling itself periodically up from the seat, thereby modulating the effective gravitational constant or by a periodic modulation of its moment of inertia at twice the swinging frequency. This proposal has given rise to the field of nonlinear optics with applications such as optical parametric oscillation (2) and the generation of entangled photon pairs by spontaneous parametric down-conversion (2–4). The last two examples require an external driving agent (“pump”) which oscillates at a frequency larger than those of the excited vibrations. The class of vibrations which I propose here is powered by a “zero”-frequency (time-invariant) force such as that of water, wind, or a DC electric field. This crucial difference, which moves the occurrence of the oscillation outside the exclusive confines of the laboratory, can be traced back, in the mathematical model, to an assumed parametric nonlinearity. The common occurrence in nature of zero-frequency forces, such as those due to wind or water, renders this class of vibrations relevant to structural stability concerns (e.g., bridges, buildings) as well as of interest to energy harvesting. These issues are commented upon in the *Discussion* section.

## Theory

To keep the theoretical development which follows on familiar and specific grounds, I chose to frame the discussion of the proposed bimodal oscillation in terms of the setting and parameters of the home shower environment where I first encountered it. The theoretical model, however, should apply broadly to all the members of the class. The model should be able to predict the following key shower observations: 1) an oscillation threshold involving the water flow rate as well as the oscillation intensity, 2) an entangled, common-frequency, vibration of the partnering modes, and 3) an unstable growing-amplitude oscillation above threshold. The freely suspended shower head has its water-ejecting surface parallel to an adjacent shower wall. With no water flowing, it can engage in two independent oscillations: the pendulum excursion $\eta \left(t\right)$ parallel to the wall and the angular twist angle $\vartheta \left(t\right)$ about the tube axis. With the water flowing, the dynamic variables assume equilibrium steady state values A and B . Denoting the time-dependent deviations from the equilibrium values by η and ϑ , so that $\eta \left(t\right)=A+\eta$ , $\vartheta \left(t\right)=B+\vartheta$ , and applying standard perturbation procedures to the Newtonian equations of motion leads to the dynamical set of coupled differential equations from which the equilibrium values A and B have been removed:

[1] $$m\frac{d^{2}\eta }{dt^{2}}+\frac{\mathrm{mg}}{l}\eta +F_{\eta }\frac{d\eta }{\mathrm{dt}}=W_{H_{2}O}\;\sin\;\vartheta ,$$

[2] $$M\frac{d^{2}\vartheta }{dt^{2}}+K_{t}\vartheta +F_{t}\frac{d\vartheta }{\mathrm{dt}}=0.$$

$F_{\eta }\;\mathrm{and}\;F_{t}$  are positive constants accounting for the dissipation of the respective modes. m  and M  are the mass and moment of inertia of the shower head, respectively, g  is the gravitational constant, and l  is the length of the water-feed tube. $K_{t}$  is the restoring torque coefficient of the water-feed tube,$W_{H_{2}O}$  is the reaction force of the forwardly ejected water which is directed oppositely to the direction of the ejected water, thus, perpendicularly to the shower head face. In Eq. **1**, I made the usual small-angle pendulum approximation, $\frac{\eta }{l}\ll 1$ , and in the remainder of this report will replace sinϑ by ϑ . With the water flow “interrupted,” $W_{H_{2}O}=0$ , η , and ϑ are decoupled, and any initial excitation decays back to zero. With the water flowing, a torsional deviation ϑ results in a force, $W_{H_{2}O}\vartheta ,$ parallel to the wall, which according to eq. 1 drives the pendulum excursion η . Assuming:

[3a] $$\eta =Re\left\{\eta_{o}e^{i\omega t}\right\}.$$

[3b] $$\vartheta =Re\left\{\vartheta_{o}e^{i\omega t}\right\}.$$

(Absent an externally imposed time reference, I am free to choose $\vartheta_{o}$ as a real number and denote the phase delay between ϑ and η by ϕ).

[4a] $$\eta =\left|\eta_{o}\right|cos\left(\omega t+\phi \right).$$

[4b] $$\vartheta =\vartheta_{o}cos\left(\omega t\right).$$

Substituting Eq. **4** in Eq. **1**, defining $\omega_{\eta }^{2}=g/l$ , and taking *d/dt* =iω , transforms [**1**] to

[5] $$m\left(\omega_{\eta }^{2}-\omega^{2}+i\frac{\omega F_{\eta }}{m}\right)\eta_{o}=W_{H_{2}O}\vartheta_{o}.$$

which is a statement of the manner by which the pendulum excursion η   , in the presence of a water flow, is driven directly by the torsional oscillation ϑ. Eqs. **1** and **2**, however, provide no clue as to the reverse process—the driving of the torsional oscillation ϑ by the pendulum excursion η . Moreover, a solution of [**2**] with $F_{t}>0$ (real dissipation) leads to an exponentially declining sinusoid:

[6] $$\begin{array}{c} \vartheta \left(t\right)=\vartheta \left(0\right)exp\left(-\frac{F_{t}}{2M}t\right)\exp\left[\pm i\omega_{t}\sqrt{1-{\left(\frac{F_{t}}{2M\omega_{t}}\right)}^{2}}t\right], \end{array}$$

where $\omega_{t}^{2}\equiv \frac{K_{o}}{M}.$ When $F_{t}$ is positive, the decaying resonance solution [**6**] is contrary to the observation in the shower of an increasing-amplitude torsional oscillation above a threshold of water force. To resolve this impasse, I resort to Lord Rayleigh/Michael Faraday’s proposal and demonstration [**1**] of an excitation of a “vibration” by a modulation of an oscillation parameter at twice the resonance frequency. A reasonable candidate parameter for this modulation is the torsional spring constant, $K_{t},$ in Eq. **2** which I take in the model to depend on the pendulum excursion $\eta \left(t\right)$ according to:

[7] $$K_{t}=K_{o}+K_{1}\eta \left(t\right)+K_{2}\eta^{2}\left(t\right)+\cdots$$

The nonlinear quadratic dependence of the last term in Eq. **7** generates the second harmonic parametric modulation (Eq. **8**) which, as shown by Rayleigh and Faraday (1), can drive the torsional oscillation. The aforementioned scientists had to import the second harmonic modulation from the “outside” to drive their single-mode oscillator. The nonlinearity of Eq. **7**, combined with the two-mode nature of the proposed class, generates the second harmonic parametric modulation needed to drive the torsional oscillation in-house and, thus, allows a zero-frequency force, $W_{H_{2}O}$ , to drive the bimodal oscillation. The assumed parametric dependence [**7**], thus, constitutes the cornerstone of the theoretical model. Experiment #3 of the Experiments section provides a direct support for its validity. (I do not attempt to explain the physical origin of the parametric dependence [**7**]. Few parametric relationships in nature are ever truly linear, and Taylor expansion approximations such as Eq. **7** are common. A good example is the second-order dielectric susceptibilities of nonlinear optics which play a role equivalent to that of $K_{2}$ in Eq. **7** (2).

Assuming a sinusoidal $\eta \left(t\right)$ as in Eq. **4** makes it possible to rewrite [**7**] in a form which brings out explicitly the in-house second harmonic parametric modulation:

[8] $$\begin{array}{c} K_{t}=K_{o}+K_{1}\left|\eta_{o}\right|cos\left(\omega t+\phi \right)\\ \;+\frac{1}{2}K_{2}{\left|\eta_{o}\right|}^{2}\left[1+cos2\left(\omega t+\phi \right)\right]+\cdots , \end{array}$$

which when substituted in Eq. **2**, leads to

[9] $$\begin{array}{c} \left(\omega_{t}^{2}-\omega^{2}+i\frac{\omega F_{t}}{M}\right)\frac{\vartheta_{o}}{2}e^{i\omega t}+c.c.\\ \;+\frac{K_{2}{\left|\eta_{o}\right|}^{2}}{4M}\left[1+\frac{e^{i(2\omega t+2\phi )}+c.c.}{2}\right]\left(\frac{e^{i\omega t}+e^{-i\omega t}}{2}\right)\vartheta_{o}=0. \end{array}$$

The retention of negative frequencies in Eq. **9** is mandated by the nonlinearity. If the negative frequencies are not retained, terms such as $\frac{K_{2}{\left|\eta_{o}\right|}^{2}}{4M}e^{i2(\omega t+\varphi )}e^{-i\omega t}\vartheta_{o}$ , which are crucial, would be absent.

Grouping together the terms in Eq. **9** with a common $e^{i\omega t}$ factor results in:

[10] $$\left(\omega_{t}^{\prime 2}-\omega^{2}\right)+i\frac{\omega F_{t}}{M}+\frac{K_{2}}{8M}{\left|\eta_{o}\right|}^{2}e^{i2\phi }=0,$$

where $\omega_{t}^{\prime 2}\equiv \omega_{t}^{2}+\frac{K_{2}{\left|\eta_{o}\right|}^{2}}{M}$ and $\omega_{t}^{2}\equiv \frac{K_{o}}{M}$.

Eq. **10** can also be written as:

[10a] $$\left(\omega_{t}^{\prime 2}-\omega^{2}\right)+i\frac{\omega F_{t}}{M}\left[1-\frac{K_{2}{\left|\eta_{o}\right|}^{2}}{8\omega F_{t}}e^{i\left(\frac{\pi }{2}+2\phi \right)}\right]=0,$$

which describes an oscillator with an effective torsional dissipation parameter ${\left(F_{t}\right)}_{\mathrm{eff}}$:

[11] $${\left(F_{t}\right)}_{\mathrm{eff}}=F_{t}\left[1-\frac{K_{2}{\left|\eta_{o}\right|}^{2}}{8\omega F_{t}}e^{i\left(\frac{\pi }{2}+2\phi \right)}\right].$$

Eq. **11** demonstrates how the nonlinear parametric physics, represented by $K_{2}$   , can control the effective dissipation of an oscillating mode, the torsional ϑ   , in this case, by the oscillation of another mode ( η   ). For sufficiently large values of $K_{2}{\left|\eta_{o}\right|}^{2}$   , the effective dissipation can become negative at values of ϕ   near $-\frac{\pi }{4}$   , or $\frac{3\pi }{4}$   , when $K_{2}>0,$   ( $\frac{\pi }{4}$   or $\frac{5\pi }{4}$   when $K_{2}<0$   ). These phases can be achieved, according to Eqs. **1**, **2**, and **13**, by small variations of the oscillation frequency ω , heretofore undetermined, about $\omega_{\eta }$:

[12] $$\eta_{o}=\frac{W_{H_{2}O}/m}{\sqrt{{\left(\omega_{\eta }^{2}-\omega^{2}\right)}^{2}+{\left(\frac{\omega F_{\eta }}{m}\right)}^{2}}}e^{i\phi }\vartheta_{o},$$

[13] $$\phi =\tan^{-1}\frac{\omega F_{\eta }}{m\left({\omega^{2}-\omega }_{\eta }^{2}\right)},$$

The phase condition $\phi =-\frac{\pi }{4},$ for example, which leads to minimal losses, obtains when

[14] $$\omega^{2}=\omega_{\eta }^{2}-\frac{\omega F_{\eta }}{m}\approx \omega_{\eta }^{2}-\frac{\omega_{\eta }F_{\eta }}{m},$$

The approximation is justified by the fact that according to Eq. **12**, the greater part of the phase shift is contributed by small changes of $\omega^{2}$ within the narrow “bandpass” (high Q-factor) Lorentzian response (5) $\left(F_{t}/M>10^{F_{\eta }}/m\right)$ . This allows the replacement of ω in Eq. **10a** by $\omega_{\eta }$ , resulting in the bimodal oscillation threshold condition:

[15] $$\left({\omega^{\prime }}_{t}^{2}-\omega_{\eta }^{2}\right)+i\frac{\omega_{\eta }F_{t}}{M}\left[1-\frac{K_{2}{\left|\eta_{o}\right|}^{2}}{8{\omega_{\eta }F}_{t}}e^{-i\left(\frac{\pi }{2}+\beta \right)}\right]=0,$$

$$2\phi \equiv -\left(\frac{\pi }{2}+\beta \right).$$

**Fig. 1.**
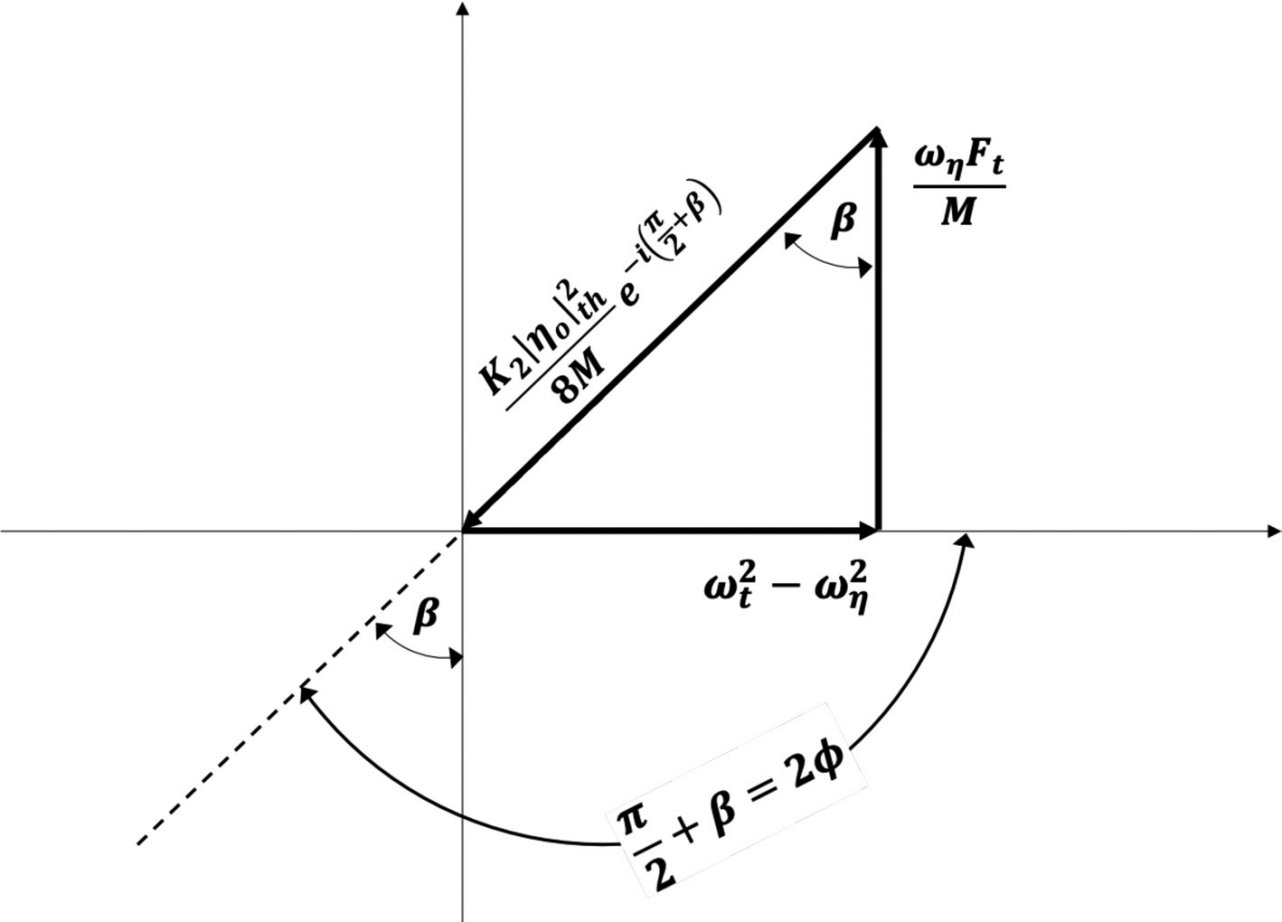
A phasor complex amplitude representation of Eq. **15**, the threshold condition for the parametric bimodal oscillation.


Eq. **15** is plotted graphically in Fig. 1. From the figure or Eq. **15**, it follows that at threshold,

[16] $$\frac{K_{2}{\left|\eta_{o}\right|}_{\mathrm{th}}^{2}}{8M}=\left(\frac{\omega_{\eta }F_{t}}{M}\right)\frac{\sqrt{{\left(\frac{\omega_{\eta }F_{t}}{M}\right)}^{2}+{\left(\omega_{t}^{2}-\omega_{\eta }^{2}\right)}^{2}}}{\frac{\omega_{\eta }F_{t}}{M}},$$

$${\left|\eta_{o}\right|}_{\mathrm{th}}^{2}=\frac{8\omega_{\eta }F_{t}}{K_{2}\cos\beta },$$

[17] $$\cos\beta =\frac{\omega_{\eta }F_{t}/M}{\sqrt{{\left(\frac{\omega_{\eta }F_{t}}{M}\right)}^{2}+{\left(\omega_{t}^{2}-\omega_{\eta }^{2}\right)}^{2}}}$$

Employing the frequency condition [**14**] in Eq. **12** leads to:

[18] $${\left|\eta_{o}\right|}^{2}=\frac{W_{H_{2}O}^{2}{\left|\vartheta_{o}\right|}^{2}}{2{\left(\omega_{\eta }F_{\eta }\right)}^{2}},\omega \approx \omega_{\eta },$$

And with the help of Eq. **17**:

[19] $${\left|\eta_{o}\right|}_{\mathrm{th}}^{2}=\frac{8\omega_{\eta }F_{t}}{\left|K_{2}\right|\cos\beta },$$

Equating [**18**] to [**19**] leads to the central result of this study, the threshold condition for the parametric bimodal oscillation:

[20] $${W_{H_{2}O}\left|\vartheta_{o}\right|}_{\mathrm{th}}=4{\omega_{\eta }}^{\frac{3}{2}}F_{\eta }\sqrt{\frac{F_{t}}{\left|K_{2}\right|\cos\beta }},$$

In the Experiments sections which follow, I compare the predictions of Eq. **20** to observations in the shower.

## The Experiments

The three video-linked experiments are designed to check the validity of the parametric assumption [**7**] to which I alluded above as the “cornerstone of the theoretical model”. Experiments 1 and 2 do so indirectly by checking the predicted dependencies of the central result: the threshold condition Eq. **20**. From Eq. **20**, it follows that at threshold, the water force $W_{H_{2}O}$   can be traded for an initial torsional oscillation amplitude $\vartheta_{o}$   and vice versa. This feature is demonstrated experimentally in Movies S1 and S2. According to Eq. **20**, the threshold condition can be reached by keeping the initial twist $\vartheta_{o}$ a constant and increasing the water force (experiment 1) or the reverse where the water force is maintained at the same level while the initial twist $\vartheta_{o}$ is increased till the oscillation threshold is reached (experiment 2). A direct support for Eq. **7** is provided by experiment 3.

### Experiment 1 (Movie S1).

At minute 00:06 , a moderate water flow is established (the rate is measured by the push-away distance of the shower head from the wall). At minute 00:09 , the shower head is twisted by ${\vartheta_{o}=45}^{o}$ . No sustained oscillation takes place. At minute 00:15 , the water flow rate is doubled and the shower head is twisted, again, by ${\vartheta_{o}=45}^{o},$ which leads to an increasing-amplitude (unstable) oscillation. These results are in agreement with the threshold expression [**20**].

### Experiment 2 (Movie S2).

The experiment is designed to show the dependence of the threshold on the initial twist $\vartheta_{o}.$ The water flow rate and thus the force $W_{H_{2}O}$ are maintained at a constant level throughout the experiment. With an initial twist angle of ${\vartheta_{o}=45}^{o}$ , no oscillation takes place. The initial $\vartheta_{o}$ is doubled to $90^{o}$ at minute 0:09 . The shower head breaks into a vigorous, increasing-amplitude vibration.

### Experiment 3 (Movie S3).

The experiment is designed to check directly the validity of the assumed parametric dependence

[7] $$K_{t}=K_{o}+K_{1}\eta \left(t\right)+K_{2}\eta^{2}\left(t\right)+\cdots$$

which I have termed the cornerstone of the theoretical model. Experiments 1 and 2 provide indirect support. A more direct confirmation would be to modulate $\eta \left(t\right)$ by an outside agent at frequencies near the fundamental torsional resonance and depend on the nonlinear term $K_{2}\eta^{2}\left(t\right)$ to generate the second harmonic needed according to Rayleigh/Faraday (1) to drive the torsional oscillation $\vartheta \left(t\right).$ The external agent in the experiment is my right hand. I move the hand left and right parallel to the wall, which enables me to control the amplitude $\eta_{1}$ and the (radian) frequency ( $\omega_{x})$ of the pendulum excursion to generate an excursion.

[E3-1] $$\eta \left(t\right)=\eta_{1}\cos\left(\omega_{x}t\right),$$

My hand motion cannot reproduce the sinusoid [Eq. **E3-1**] but can generate a reasonable approximation of a periodic excursion $\eta \left(t\right)$ with a controlled repetition (radian) frequency $\omega_{x}.$ $\eta_{1}$ is the amplitude of the first term (fundamental) of the Fourier series expansion of the periodic hand-generated excursion.

The analysis of this scenario exists already in the development leading up to (9) which is rewritten in what follows (with a slight labeling alteration):

[E3-2] $$\begin{array}{c} \left(\omega_{t}^{2}-\omega^{2}+i\frac{\omega F_{t}}{M}\right)\vartheta_{o}e^{i(\omega t-\phi )}+c.c.\\ \;=-\frac{K_{2}{\left|\eta_{1}\right|}^{2}}{2M}\left[1+\frac{e^{i2\omega_{x}t}+c.c.}{2}\right]\left[\frac{e^{i\left(\omega t-\phi \right)}+e^{-i\left(\omega t-\phi \right)}}{2}\right]\vartheta_{o}, \end{array}$$

For Eq. **E3-2** to be satisfied, it is required that $\omega_{x}=\omega$ , so that the torsional oscillation frequency ω is that of the hand excursion. Grouping together terms with an $exp(i\omega_{x}t)$ dependence results in:

[E3-3] $$\left[\omega_{t}^{'2}-\omega_{x}^{2}+\frac{K_{2}\eta_{1}^{2}}{4M}+i\frac{\omega_{x}F_{t}}{M}\right]+\frac{K_{2}\eta_{1}^{2}}{8M}e^{i2\phi }=0,$$

The threshold condition for oscillation $\eta \left(t\right)$ is thus given by

**Fig. 2.**
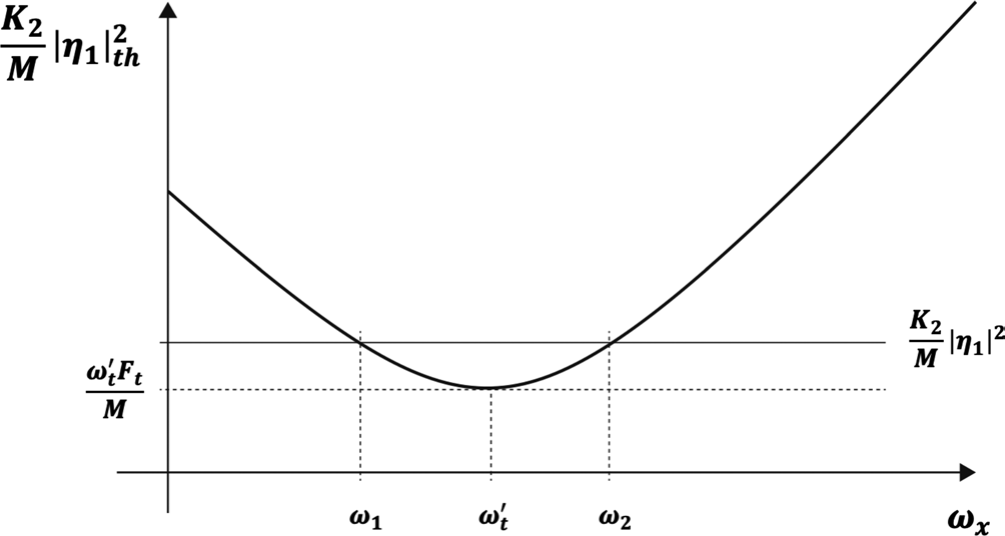
The threshold condition for parametric oscillation of the torsional mode ϑ(t) due to an externally generated pendulum excursion $\eta_{x}\left(t\right)=\eta_{1}\cos\left(\omega_{x}t\right).$

[E3-4] $${\left|\eta_{1}\right|}_{\mathrm{th}}^{2}=\left(\frac{8M}{K_{2}}\right)\sqrt{{\left(\omega_{t}^{\prime 2}-\omega_{x}^{2}\right)}^{2}+{\left(\frac{\omega_{x}F_{t}}{M}\right)}^{2}},$$

and is plotted in Fig. 2.

The solid dark curve is a plot of Eq. **E3-4**, the oscillation threshold value of the externally driven ${\left|\eta_{1}\right|}^{2}$ . The horizontal solid line is the value of the pendulum excursion amplitude (squared) controlled by my hand motion which I maintain, reasonably, constant in the experiment. The oscillation condition ${\left|\eta_{1}\right|}^{2}>{\left|\eta_{1}\right|}_{\mathrm{th}}^{2}$ is satisfied for frequencies between $\omega_{1}$ and $\omega_{2}$ . The relevant parameters which I measured in the shower with an iPhone are $F_{t}/M≅2s^{-1},$ $\omega_{t}^{\prime }≅12s^{-1}$ . In the experiment, I increase $\omega_{x}$ in three stages from zero to a high value, $\omega_{x}\gg \omega_{t}^{\prime }$.

The experiment can be observed in linked video clip #3. Between 00:00 and 00:06 , the hand-controlled frequency satisfies $\omega_{x}<\omega_{1}$ , where according to Fig. 2 or, equivalently, Eq. **E3-4** ${\left|\eta_{1}\right|}^{2}<{\left|\eta_{1}\right|}_{\mathrm{th}}^{2}$ and no torsional oscillation takes place. Vigorous torsional oscillation is observed between minutes 00:06 and 00:13 where ${\omega_{1}<\omega }_{x}<\omega_{2}\;\mathrm{and}\;{\left|\eta_{1}\right|}^{2}>{\left|\eta_{1}\right|}_{\mathrm{th}}^{2}$ . At minute 00:13 , $\omega_{x}$ has been increased so that $\omega_{x}>\omega_{2},$ where ${\left|\eta_{1}\right|}^{2}<{\left|\eta_{1}\right|}_{\mathrm{th}}^{2}$ and no torsional oscillation takes place. These observations are in qualitative agreement with Eq. **E3-4** as plotted in Fig. 2. A residual “small” water flow was kept to serve as a visual angular marker of the twist angle $\vartheta \left(t\right)$ and did not affect the conclusions.

## Discussion

The bimodal oscillation described above is inherently unstable. Once threshold is exceeded, the exponential growth coefficient keeps increasing with the oscillation level ${\left|\eta_{o}\right|}^{2}$   . The instability is evident in the Movies S1 and S2 and is traceable mathematically to the fact that the key parametric nonlinearity responsible for driving the torsional oscillation is represented, as in Eqs. **10a** and **11**, by $\frac{K_{2}}{8M}{\left|\eta_{o}\right|}^{2},$ which keeps increasing above threshold even as the water force is kept constant. An equivalent point of view is that the effective dissipation, ${\left(F_{t}\right)}_{eff,}$ in Eq. **11** decreases with an increase in the oscillation level ${\left|\eta_{o}\right|}^{2}$ and becomes negative. This instability will need to be controlled in energy-harvesting applications. One possible solution would be to engineer the modal dissipation parameters $F_{\eta }$ or $F_{t}$ , or both, so as to increase with the oscillation level. This will play a role similar to that of the gain (amplification) in laser oscillators where the exponential gain coefficient decreases with increasing optical intensity by a saturation of the population inversion. This essential instability can, for example, channel the, practically limitless, zero-frequency energy of a wind into an increasing-amplitude resonant oscillation of a structure such as a building or a bridge. A key to the destructive potential of the bimodal oscillation proposed herein is that it acts as a spectral translator moving effectively the driving force energy from the steady, zero frequency to that of twice resonance where, depending on the modal dissipation parameters, its effect is amplified (by more than an order of magnitude in the shower experiment). This resonance-seeking self-tuning is enabled by the nonlinear parametric dependence [**7**] and takes place automatically since it leads to the lowest threshold conditions.

Eyewitness and video accounts of the Tacoma Narrows bridge collapse in 1940 during a windstorm (5) describe a dual, horizontal, and vertical, oscillation of the bridge roadbed culminating in the collapse. The sharing of the key features—bimodality, a threshold, instability, and a steady driving force (wind, water)—by both oscillations in the bridge collapse and in the shower oscillation should make the parametric bimodal oscillation model advanced herein of interest to structural design considerations.

## Supplementary Material

Appendix 01 (PDF)

Movie S1. The tradability of water force, W_H_2_O_, in the threshold relation (eq.20) for an initial twist angle, 𝜗_o_. Twist angle remains constant.

Movie S2. The tradability of water force, W_H_2_O_, in the threshold relation (eq.20) for an initial twist angle, 𝜗_o_. Water force remains constant.

Movie S3. Manual parametric driving of the torsional oscillation.

## Data Availability

All study data are included in the article and/or supporting information.
