# Supplementary material for: On a class of bimodal oscillations powered by a steady, zero-frequency force—Implications to energy conversion and structural stability
Source: Proc Natl Acad Sci U S A. 2023 Sep 11;120(38):e2311412120. doi: 10.1073/pnas.2311412120 (PMC10515148; doi:10.1073/pnas.2311412120)
Supplement: Supplementary file 1 — Appendix 01 (PDF) [file pnas.2311412120.sapp.pdf]

Video Clip Legends

Video Clip #1 (Experiment #1): The tradability of water force, W_{H_2O}, in the threshold relation (eq.20) for an initial twist angle, ϑ_o. Twist angle remains constant.

Video Clip #2 (Experiment #2): The tradability of water force, W_{H_2O}, in the threshold relation (eq.20) for an initial twist angle, ϑ_o. Water force remains constant.

Video Clip #3 (Experiment #3): Manual parametric driving of the torsional oscillation.
